# The Book World of Medicine and Science

**Published:** 1907-07-06

**Authors:** 


### The Book World of Medicine and Science.

International Clinics. Sixteenth Series, Vol. IV.; and Seventeenth Series, Vol. I. (Philadelphia and London: J. B. Lippincott Company. 1906-7.)

We have quite recently had an opportunity of expressing our appreciation of the high standard maintained in this valuable serial publication. The two issues now before us are fully up to the level of their predecessors. They provide a most excellent list of original articles and clinical lectures on subjects included in the various departments of medicine, and we have no hesitation in commending them to our readers. In the last volume for the year 1906 may be found a convenient and lucid statement of medical progress during the preceding twelve months.

